# Supplementary material for: An empirical study on KDIGO-defined acute kidney injury prediction in the intensive care unit
Source: Bioinformatics. 2024 Jun 28;40(Suppl 1):i247–56. doi: 10.1093/bioinformatics/btae212 (PMC11211814; doi:10.1093/bioinformatics/btae212)
Supplement: btae212_Supplementary_Data [file btae212_supplementary_data.pdf]

A. Supplementary results

A.1. Prediction performance by length of ICU stay

Motivated by the high importance of time-since-admission for the GBDT model, we compared the performance of both models for patients with different categories of length-of-stays. We observe better performance of both models in longer stays, however for the GBDT model the performance drop is smaller than for the LSTM model.

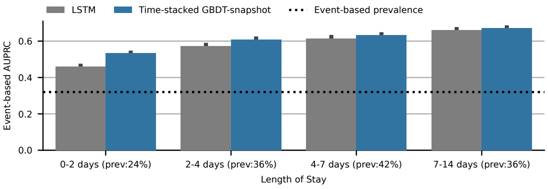

Supplementary Figure 1. Model performance in subcohorts with different lengths of stay in the ICU. The AUPRCs are prevalence-corrected. Performance in the HiRID-II test set is shown.

A.2. Comparison with a clinical baseline

While the performance of clinicians in the AKI prediction task can only be assessed using a clinical study, we tried to approximate rule-based clinical decision making for the AKI with a simple decision tree (16 leaves) using the 3 most clinically relevant variables for AKI prediction: creatinine, urine output as well as dialysis status. We observe that the best-performing GBDT model significantly outperforms such a decision tree.

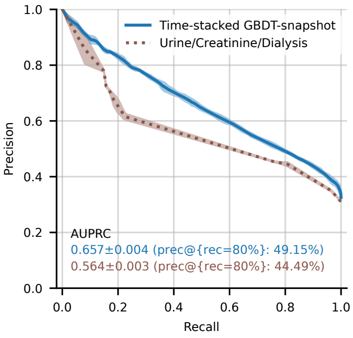

Supplementary Figure 2. Comparison between best performing GBDT model and a baseline model that mimics rule-based decision making in an ICU. Performance in the HiRID-II test set is shown.

B. Supplementary methods

B.1. Details on partial imputation

We partially impute data on a time grid with a step size of 5 minutes between the first and last heart rate observation. Data was cut off after 28 days, to avoid abnormally long stays biasing the modeling process. Partial imputation means that data was filled with plausible values for some grid points, but left missing for others where a current value could not be estimated. Using prior clinical knowledge, for each parameter an imputation mode was defined. We distinguish between

- **In-definite forward filling**: Data is filled from the last observation until every succeeding time grid point.
- **Limited forward filling**: Either using prior clinical knowledge, or estimated from the data, we defined a maximum time period for which the last measurement was forward filled on the time grid. For the latter we obtained the distribution of measurement intervals in the training set, we then forward fill for up to $2 * \text{median(interval)} + \text{std(interval)}$ minutes.
- **No forward filling**: Data is not forward filled, only the grid points closest to the observations have a value.

B.2. Details on feature engineering for GBDTs

We extracted several types of features at each grid point, which we summarize below. Feature engineering takes partially imputed data as specified above as input. Only the `GBDT-history` types of model use features beyond the snapshot values.

- **Snapshot value**: The partially imputed value at a grid point is used directly as a feature.
- **Static data**: Static information available at the beginning of the ICU stay is concatenated to the feature vector, such as gender, patient age, etc.
- **History summary**: For each clinical parameter, we use 4 horizons, corresponding to the 20 %, 40 %, 60 %, 80 % percentiles of available history at a randomly sampled time-point of an ICU stay. This corresponded to 10, 26, 63, 156h respectively. For continuous variables, mean, standard deviation and trend over the horizons were computed, for categorical variables the mode, and for binary variables the mean was used.
- **Measurement density**: The time to the last measurement as well as the density of measurement in the 4 horizons specified above were computed.

B.3. Re-implementation of the LSTM-based model by Tomasev et al.

Here we present an in-depth discussion of our LSTM model, a modernized reimplementation of the original model described by Tomašev et al. (2019). Our version, developed using PyTorch, includes several key modifications to enhance its performance and capabilities.

The decision to reimplement the LSTM model in PyTorch was driven by several factors. Primarily, the original implementation was based on TensorFlow 1.X, which was superseded in 2019 and is less readable compared to more modern frameworks like Tensorflow 2.X or Pytorch 2.X. This change to PyTorch 2 not only enhances readability but also ensures the longevity and maintainability of the code. Additionally, the GitHub repository for the original model has been archived, suggesting a lack of ongoing support and updates for the codebase. Another significant factor in our decision was the data representation format. The original model used protocol buffers to represent patients' medical records, a format that is not widely adopted in the machine learning community. In contrast, our implementation uses torch tensors or numpy arrays, aligning with standard practices in the field.

Our model exhibits several key differences from the original implementation by Tomašev et al. (2019). In terms of model structure, while the original implementation includes a cumulative distribution function layer accommodating multiple prediction horizons (see Extended Data Fig. 2 of Tomašev et al. (2019)), our model is tailored to predictions for a

single horizon. Preliminary experiments indicated that the inclusion of multiple horizons did not improve the prediction performance.

Regarding feature engineering, there are noteworthy differences. The original model aggregated data into a 6-hour grid, incorporating a variety of summary statistics within each interval. In contrast, our model uses simpler features without such aggregation, owing to the 1-hour resolution data available in the MIMIC-IV data set. Furthermore, Tomašev et al. (2019) included the construction of high-level features, like the occurrence of groups of variables within each 6-hour bucket. Our implementation does not follow this approach, primarily because our data set consists of only 28 variables, which simplifies the process of feature engineering. In addition to the 28 variables, we also constructed binary presence indicators for all variables at each time step, to enable our models to distinguish between the absence of a numerical value and an actual value of zero. Regarding the auxiliary prediction task of predicting the maximum future observed value of a set of variables over the same horizon that we used to make the future AKI predictions (i.e., 48 hours), we selected the following target variables guided by ICU clinicians: serum creatinine, accumulated urine output, fluid input and loop diuretics.
